# Beyond PET/CT in Hodgkin lymphoma: a comprehensive review of the role of imaging at initial presentation, during follow-up and for assessment of treatment-related complications

**DOI:** 10.1007/s13244-015-0407-z

**Published:** 2015-04-28

**Authors:** Abhishek R. Keraliya, Sree Harsha Tirumani, Atul B. Shinagare, Nikhil H. Ramaiya

**Affiliations:** Department of Radiology, Harvard Medical School, Brigham and Women’s Hospital, 75 Francis Street, Boston, MA 02115 USA; Department of Imaging, Harvard Medical School, Dana Farber Cancer Institute, 450 Brookline Avenue, Boston, MA 02215 USA

**Keywords:** Hodgkin lymphoma, PET/CT, Computed tomography, MRI ultrasound

## Abstract

**Objective:**

The purpose of this article is to provide a comprehensive review of the role of imaging modalities other than PET/CT in the management of Hodgkin lymphoma (HL). PET/CT is the imaging modality of choice in the management of Hodgkin’s lymphoma (HL). However, imaging modalities other than PET/CT such as plain radiographs, ultrasound, CT, MRI and nuclear imaging can help in various stages of clinical management of HL, including the initial workup and post-treatment surveillance. Both CT and MRI help in detecting recurrences, treatment-related pulmonary, cardiovascular and abdominal complications as well as second malignancies. Familiarity with expected post-treatment changes and complications on surveillance images can help radiologists guide patient management. The purpose of this article is to provide a comprehensive review of the role of imaging modalities other than PET/CT in the management of Hodgkin lymphoma (HL).

***Main Messages*:**

• *Surveillance of HL patients is usually performed with plain radiographs and CT.*

• *Follow-up imaging can depict normal post-treatment changes or treatment-related complications.*

• *Imaging is important for the timely detection of second malignancies in HL patients.*

## Introduction

Hodgkin lymphoma (HL) accounts for approximately 10 % of all lymphomas diagnosed in the developed world annually [1]. The WHO classification divides HL into two main types: classical HL (CHL) and nodular lymphocyte predominance HL (NLPHL). CHL is further subdivided into four histological subtypes: nodular sclerosis CHL (NSCHL), mixed cellularity CHL (MCCHL), lymphocyte-rich CHL (LRCHL) and lymphocyte-depleted CHL (LDCHL) [2]. HL has bimodal age distribution with most patients diagnosed between 15 and 30 years of age, followed by another peak in adults over the age of 50. HL is usually confined to the lymph nodes. Cervical and mediastinal lymph nodes are the most common site of lymph nodal involvement in HL. In mediastinal lymph nodes, prevascular and paratracheal subsets are most commonly affected. Isolated infradiaphragmatic lymphadenopathy occurs in less than 10 % of patients at diagnosis [3]. Extranodal involvement is less common in HL than in NHL and seen in only 10–15 % of patients. The most frequent sites of extranodal involvement are bone, bone marrow, lung and liver [4].

HL is now curable in more than 80 % of cases owing to significant therapeutic advances in the form of newer chemotherapeutic drugs, advances in radiotherapy and stem cell transplantation [5]. Treatment for HL usually depends on the stage at diagnosis, histological subtype and prognostic factors. According to National Comprehensive Cancer Network (NCCN) guidelines, the initial evaluation of patients with HL includes detailed history and physical examination, evaluation of performance status, laboratory investigations (blood counts, ESR, LDH, liver and renal function tests) and radiological investigations (chest radiograph, PET/CT, contrast-enhanced CT). Staging for HL is based on the Cotswolds modification of the Ann Arbor staging system.

Patients with HL are usually classified into four groups: early-stage favourable (stage I–II with no unfavourable factors), early-stage unfavourable (grade I–II with any of the unfavourable factors), advanced favourable (clinical stage III or IV with zero to three adverse risk factors listed below) and advanced unfavourable (clinical stage III or IV with four or more adverse risk factors listed below). Large mediastinal adenopathy (>33 % of the thoracic width on the chest x-ray, ≥10 cm on CT), presence of B symptoms, more than 2 or 3 nodal sites of disease or an ESR of 50 or more are unfavourable prognostic factors for patients with stage I and II disease. Assessment of prognosis is important for formulating management strategies.

For patients with advanced-stage HL, the International Prognostic Factors Project has developed an International Prognostic Index with a prognostic score that is based on the following seven adverse factors: age more than 45 years, male gender, stage IV disease, albumin level below 4.0 g/dl, haemoglobin level below 10.5 g/dl, white blood cell count more than 15,000/mm³, absolute lymphocytic count less than 600/mm³ or a lymphocyte count less than 8 % of the total WBC count [6].

Chemotherapy or combination chemotherapy plus low dose involved field radiation therapy (LD-IFRT) is the standard treatment option in HL. The most common chemotherapeutic regimes used for HL are ABVD (doxorubicin, bleomycin, vinblastine, dacarbazine), Stanford V (doxorubicin, vinblastine, mechlorethamine, vincristine, bleomycin, etoposide and prednisone) and BEACOPP (bleomycin, etoposide, doxorubicin, cyclophosphamide, vincristine, procarbazine and prednisone). Patients with nonbulky stage IA or IIA disease are considered to have clinical early-stage disease. These patients are candidates for chemotherapy, combined modality therapy or radiation therapy alone [7]. The number of cycles is determined by the pre-treatment prognostic factors (favourable or unfavourable disease) and the rate of response to treatment. The treatment for HL also depends on the patient’s performance state and the response to treatment. ABVD, Stanford V or escalated BEACOPP is the preferred therapy for patients with advanced HL. For patients with refractory HL or who relapse after chemotherapy and involved-field radiation therapy, second-line chemotherapeutic agents, biological therapy (such as rituximab) and autologus stem cell transplant are treatment options. The role of consolidation radiotherapy after chemotherapy in patients with advanced HL is controversial; however involved-field radiotherapy (IF-RT) is beneficial for patients with partial response after chemotherapy according to a prospective randomised trial of 739 patients with advanced HL [8].

Due to improved long-term survival, treatment-related complications have emerged as an important cause of morbidity and mortality in patients with HL. Early and accurate diagnosis of treatment-related cardiovascular and pulmonary complications and second malignancies are of paramount importance to continue the survival benefit in these patients, especially in those treated with mantle field radiation.

Imaging, especially PET/CT, plays a key role in the diagnosis, staging, response assessment, prognostication and surveillance of patients with HL. Though FDG-PET/CT is the imaging modality of choice in HL, other modalities such as conventional radiographs, ultrasound (US), CT and magnetic resonance imaging (MRI) are often used in the evaluation of lymphoma at various stages of management. In this article, we review the role of various imaging modalities beyond FDG-PET/CT in the management of HL with emphasis on imaging features of treatment-related complications.

## PET/CT in Hodgkin’s Lymphoma

According to NCCN, 18 F-Fluoro-DeoxyGlucose (FDG) positron emission tomography (PET)/computed tomography (CT) is the corner stone for initial staging and evaluation of treatment response in HL. Response to treatment with chemotherapy or combined modality therapy is assessed by restaging with FDG-PET/CT at midcycle (interim PET/CT) or completion of treatment. FDG-PET positivity at the treatment completion is an adverse prognostic factor for disease-free survival [9]. Metabolic imaging with PET often leads to either upstaging or downstaging in approximately 15–40 % of patients with Hodgkin lymphoma with impact on management in about 5–15 % [10, 11]. Several studies have established the role of interim FDG-PET in the prognosis of HL during chemotherapy. PET is particularly useful in the evaluation of a post-treatment residual mass in patients after the completion of therapy, with positive FDG-PET associated with a significantly poorer progression-free survival [12]. Negative interim FDG-PET/CT is highly predictive of progression-free survival, even more than the International Prognostic Score (IPS) [13, 14]. According to a prospective study, the sensitivity and specificity of FDG PET to predict relapse are 79 % and 97 %, respectively, with a negative predictive value more than 90 % in HL patients after the completion of chemotherapy [15]. Negative PET study reliably rules out disease relapse. However, the positive predictive value of PET is variable, which is an important limitation of PET, and a biopsy is necessary to confirm the diagnosis of a relapse [16]. FDG-PET/CT is also useful in radiation treatment planning and helps in better delineation of the radiation field without increasing radiation volumes [17]. There are some important limitations of FDG-PET/CT. FDG uptake can also be seen in post-treatment inflammation, infection, brown fat and normal physiologic metabolic activity, which can lead to false-positive results. Similarly, low-grade histological subtypes and minimal residual disease can give false-negative results.

## Imaging beyond PET at initial staging

**Fig. 1:**
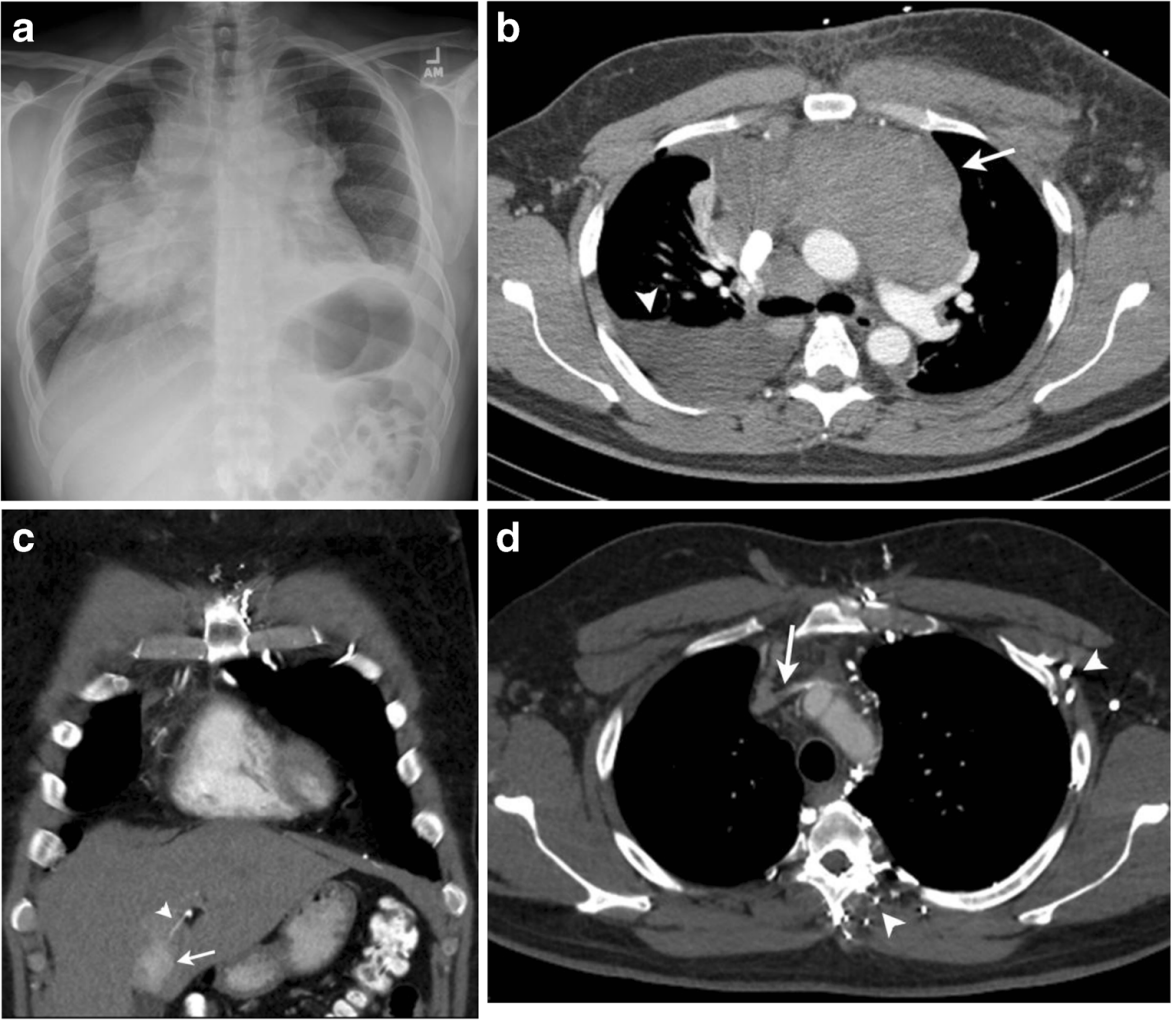
A 39-year-old male with increasing cough, night sweats and weight loss since 2 months. **a** Frontal chest radiograph demonstrates bulky mediastinal lymphadenopathy exceeding one third of the internal transverse diameter of the thorax. **b** Axial contrast-enhanced CT image reveals large conglomerate anterior mediastinal lymph nodal mass (*arrow*) and right-sided pleural effusion (*arrowhead*). Biopsy of lymph nodal mass was suggestive of nodular sclerosing HL. Follow-up CT study after treatment with chemotherapy and mediastinal radiation complicated by chronic venous thrombosis. **c** Coronal contrast-enhanced CT image shows focal hyperenhancement (*arrow*) in segment IV of the liver due to reflux in a capsular vein (*arrowhead*) communicating with the inferior phrenic vein (not shown). **d** Axial contrast-enhanced CT image demonstrates chronic occlusion of the left brachiocephalic vein (*arrow*) with multiple chest wall and paravertebral venous collaterals on the left side (*arrowheads*)

The most typical presentation of HL is painless, enlarged superficial lymphadenopathy with systemic symptoms such as fever, night sweats, fatigue and weight loss. Patients with intrathoracic lymphadenopathy may present with respiratory symptoms such as cough, dyspnoea and chest pain. Chest radiography can be performed as the initial investigation in these patients. Chest X-ray provides preliminary information about involvement of the mediastinum and lungs. Bulky mediastinal lymphadenopathy at diagnosis is an unfavourable prognostic factor in patients with HL and can be quantified by measuring using the mediastinal mass ratio. Cotswolds modification of the Ann Arbor staging system defines bulky disease as a mediastinal mass exceeding one third of the internal transverse diameter of the thorax at the T5/6 intervertebral disc level on a posteroanterior chest radiograph (Fig. 1) [18]. US is often helpful in confirming enlarged nodes and guiding biopsy, particularly cervical lymph nodes, the most common nodal group affected in patients with HL. US can be useful for evaluation of extranodal sites of involvement in the abdomen (e.g., gallbladder, kidney) and image-guided biopsy of focal lesions in solid organs such as the liver and spleen [19].

CT can be performed for further evaluation of abnormalities detected on chest x ray. CT can help in depicting other areas of lymph node enlargement that are not obvious on chest radiographs. On CT, HL is characterised by the presence of a discrete anterior mediastinal mass with a lobulated contour with homogeneous soft-tissue attenuation. Large lymph nodal masses can have a heterogeneous appearance with low attenuation areas representing necrosis, haemorrhage or cystic degeneration (Fig. 1). CT is particularly useful for exact anatomical delineation of lymph nodal involvement and in formulating treatment plans and radiation fields.

Although PET/CT remains the corner stone for initial staging and response assessment at the completion of treatment in patients with HL, MRI plays an important part in management of patients with HL. The role of MRI is particularly important in paediatric and younger patients because of exposure to ionising radiation in diagnostic modalities such as CT and PET/CT. In recent years, diffusion-weighted imaging (DWI) has been emerging as a radiation-free alternative to PET/CT for staging of various malignancies including lymphoma [20]. DWI is a functional MRI technique that depicts differences in the mobility of water in tissues. Hypercellular tumours exhibit more restricted water diffusion than normal tissues, which is reflected as high signal intensity on DWI with low apparent diffusion coefficient (ADC) values. DWI-MRI has sensitivity up to 97 % and is a feasible alternative to PET/CT for follow-up and treatment response assessment in patients with FDG-avid lymphoma [21, 22]. The whole body-DWI technique does not need specific equipment and is well tolerated by patients thanks to the relatively shorter acquisition time, which is longer than the acquisition time for CT but shorter than for PET/CT. The use of higher magnetic field strength (3 T) has a higher signal-to-noise ratio (SNR), which improves the ability of lesion detection. The cost of a WB-MRI is roughly equal to the cost for an 18F-FDG PET/CT examination. MRI is also useful in evaluation of intracranial disease and spinal cord involvement because of better soft tissue resolution. In the thorax, MRI is beneficial for assessment of chest wall, vascular, cardiac and pleural and brachial plexus involvement [23].

Central venous thrombosis and compression: Superior vena cava (SVC), brachiocephalic or internal jugular veins can be directly compressed by the mediastinal adenopathy with development of SVC syndrome. Lymphoma is responsible for about 10 % of malignant SVC obstruction cases [24]. SVC and brachiocephalic venous thrombosis can also complicate chemotherapy catheter placement in patients with HL. Contrast-enhanced chest CT is the modality of choice for evaluation of SVC obstruction and to differentiate between venous thrombosis and extrinsic compression. Chest wall venous collaterals and characteristic focal hyperenhancement of liver (“hot-spot sign”) is seen with SVC or brachiocephalic vein obstruction due to opening of caval-mammary-phrenic–hepatic capsule-portal pathways (Fig. 1). In this pathway, blood flows from the internal mammary vein to the inferior phrenic vein. The inferior phrenic vein then communicates with hepatic capsular veins, which in turn drain into the intrahepatic portal tributaries. Focal non-mass-like contrast enhancement is seen along the superior aspect of the liver with dilated inferior phrenic veins and hepatic capsular veins [25, 26].

**Fig. 2:**
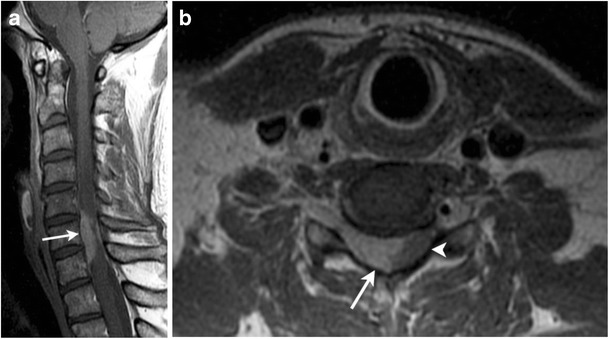
A 36-year-old male with newly diagnosed nodular sclerosing HL presenting with numbness in his right hand. **a** Sagittal contrast-enhanced T1-weighted image of the cervical spine demonstrates enhancing epidural soft tissue (*arrow*) at the C4 to C6 vertebral levels. **b** Axial contrast-enhanced T1-weighted image demonstrates epidural enhancing soft tissue (*arrow*) with significant compression and mass effect on the cervical spinal cord (*arrowhead*)

Central nervous system (CNS) involvement in HL is rare with an overall reported incidence of 0.2–0.5 % [27]. Intracranial HL is usually seen in patients with relapsed and progressive disease as a part of widespread systemic involvement. The most common histological subtypes of CHL associated with CNS involvement are MCHL and NSHL. Evaluation of CNS involvement in HL on FDG-PET is limited by the intense physiologic FDG uptake in the brain. CT with intravenous contrast and MRI are useful in such instances. Spinal cord compression is a rare complication of HL, occurring usually in the setting of progressive and advanced disease. As the initial symptom of HL, it is extremely rare, with only a few cases reported in the literature [28]. Prompt diagnosis is crucial as early surgical decompression, radiation therapy and chemotherapy are imperative to prevent irreversible neurological deficits. MR imaging of the spine, without and with the use of gadolinium-based contrast material, is the standard for the diagnosis of malignant spinal cord compression (Fig. 2) [29].

## Imaging beyond PET during follow-up

**Fig. 3:**
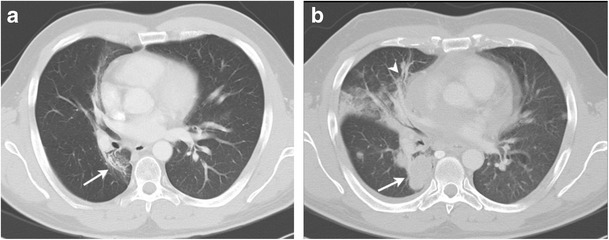
A 35-year-old male with a history of HL treated 3 years ago with chemotherapy and mediastinal radiation presenting with relapsed HL in the radiation field. **a** Axial CT image in lung window settings shows well-demarcated area of parenchymal distortion and traction bronchiectasis in a paramediastinal location in the right lower lobe (*arrow*) suggestive of post-radiation changes. **b** Axial CT image in lung window settings after 3 years shows the appearance of a lobulated mass within the radiation field with obliteration of the previously visualised air bronchogram (*arrow*). New appearance of ground-glass opacities with consolidation (*arrowhead*) is seen in the right middle lobe

Due to the long-term risks and complications of the therapies of HL, regular follow-up of treated patients with HL is important. The follow-up schedule depends on the age of patients, staging at the time of diagnosis and type of treatment given. Physical examinations and blood tests are performed every 3 to 6 months for the first 2 years, then every 6 to 12 months for the next 3 years and then annually [30]. The NCCN guidelines recommend imaging techniques such as chest radiographs or chest CT and abdomen and pelvis CT to be performed every 6 months for the first 2 years and then annually for the next 3 years. If the neck is involved in the radiation treatment field, thyroid function tests are performed annually. The overall 5-year survival rate for early-stage HL in children and adolescent population is more than 90 % [31]. However, up to 10 % of patients with early-stage and up to 25 % patients with advanced-stage HL relapse after first-line therapy [32]. In addition to detecting long-term treatment-related complications, the goal of the surveillance scans is to detect local and distant recurrences of lymphoma. Recurrent lymphoma has the same appearance as primary HL; however pulmonary parenchymal involvement is more common in recurrent HL. The various patterns of parenchymal involvement include a pulmonary nodule or mass, lobar or segmental consolidation with an air bronchogram and a reticular pattern with peribronchovascular and interlobular septal thickening [33]. Findings suggestive of recurrent disease in the radiation field include an alteration in the contour of the radiation fibrosis with an increase in size, convex borders, appearance of homogeneous opacity with obliteration of the air bronchogram and filling of the bronchi (Fig. 3) [34].

**Fig. 4:**
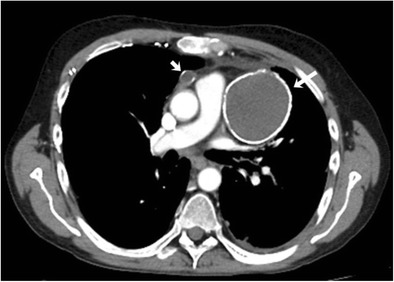
A 37-year-old male with a history of HL treated with chemotherapy and mediastinal radiation 5 years ago. Axial contrast-enhanced CT image reveals anterior mediastinal lymphadenopathy with peripheral calcification (*arrows*)

Restaging CT examinations often demonstrate findings that represent expected post-treatment changes. Dystrophic lymph nodal calcification is seen in approximately 2–8 % of treated patients with HL following radiation or chemotherapy (Fig. 4). Lymph nodal calcification in untreated patients is seen in less than 1 % of patients with aggressive histological subtypes of HL [35]. In treated patients, dystrophic calcification represents treatment-induced necrosis and is associated with good prognosis and long-term survival [36]. Absence of spleen is another finding on imaging that can be encountered in patients with HL. Before effective chemotherapy and radiotherapy were available and staging was crucial to differentiate limited-stage disease from extensive-stage disease, splenectomy was often performed in patients with HL as 20–35 % with clinically limited disease confined to above the diaphragm will have occult splenic involvement [37].

**Fig. 5:**
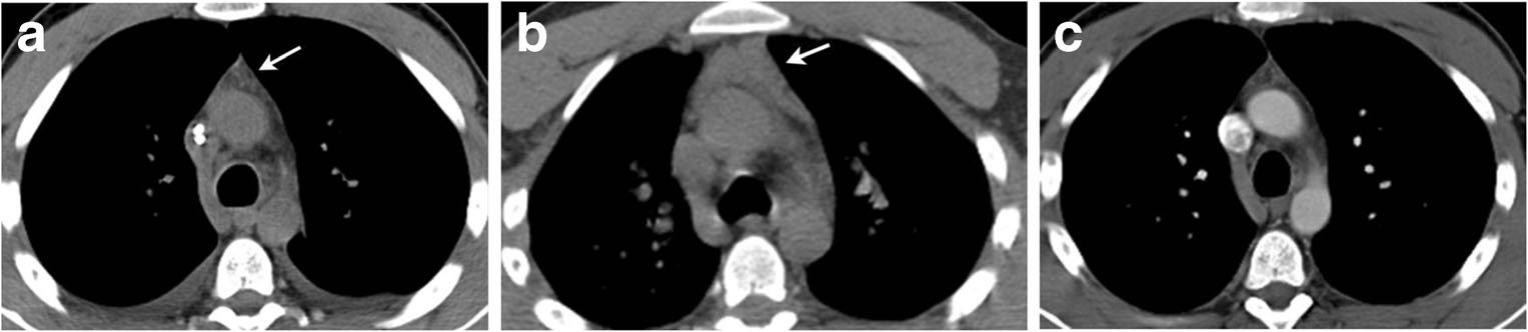
A 22-year-old male with relapsed HL shows thymic rebound after second-line chemotherapy. **a** Axial non-contrast CT image shows triangular low-attenuation anterior mediastinal soft tissue (*arrow*) suggestive of normal thymus. **b** Axial non-contrast CT image after 3 months of chemotherapy shows diffusely enlarged thymus (*arrow*). The thymic contour is maintained with smooth margins and absence of surrounding vascular invasion. **c** Axial non-contrast CT image 4 months after completion of chemotherapy shows resolution of thymic enlargement with similar appearance of thymus compared to pretreatment study

Rebound hyperplasia of the thymus occurs in 10 to 25 % of patients treated with chemotherapy [38]. Thymic rebound usually occurs 1 to 11 months after completion of chemotherapy because of reactive lymphoid hyperplasia in response to withdrawal of chemotherapy and is usually self-limiting and reversible [39]. It is important to differentiate thymic rebound from recurrent mediastinal disease. CT features that favour thymic regrowth over lymphomatous thymic involvement or thymic tumour are a diffusely enlarged thymus that maintains the normal thymic morphology with a smooth contour, uniform density without invasion of adjacent tissues and absence of pericardial or pleural effusion (Fig. 5).

**Fig. 6:**
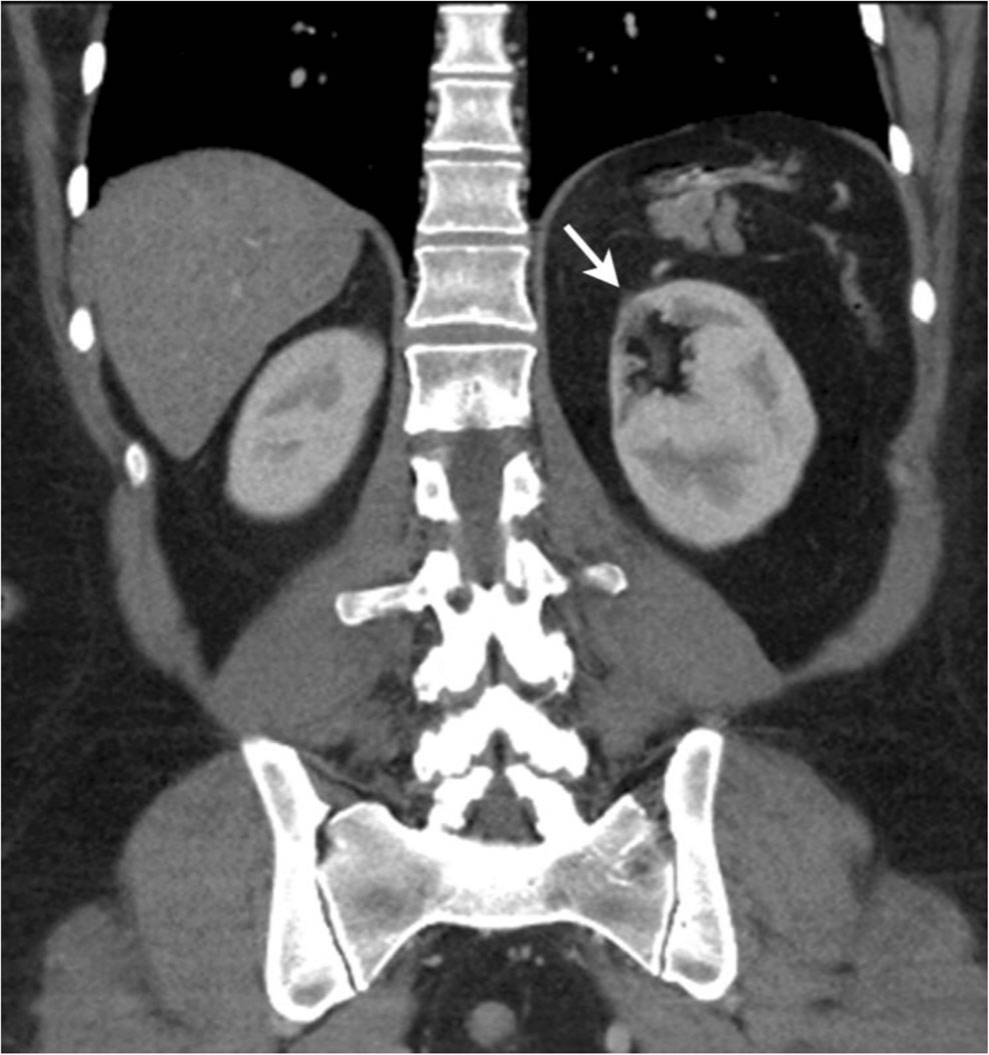
A 49-year-old female with a history of nodular sclerosing HL treated 23 years ago with mantle and paraaortic radiation to a total dose of 36 Gy. Coronal reformatted image of contrast-enhanced CT shows cortical thinning of the upper pole of the left kidney (*arrow*). Note the absence of spleen from the prior splenectomy

**Fig. 7:**
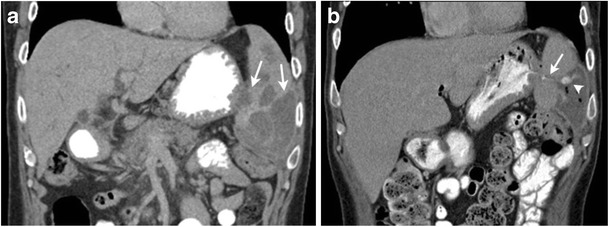
A 49-year-old male with relapsed HL with splenic lesions treated with second-line chemotherapy. **a** Coronal contrast-enhanced CT image shows splenomegaly with multiple low-attenuation splenic lesions (*arrows*). **b** Coronal contrast-enhanced CT image 4 months after treatment with second-line chemotherapy demonstrates communication between the stomach and spleen (*arrow*) with the presence of hyperdense oral contrast material (*arrowhead*) and air specs in a low-attenuation splenic lesion suggestive of gastrosplenic fistula

Until the 1990s in the prechemotherapy era, radiation therapy was often used as a single modality in the treatment of HL with more extended fields and higher doses than in current radiotherapy techniques [40]. In patients who have received infradiaphragmatic radiotherapy, various abdominal organs could be affected by side effects of radiation. Radiation-induced liver disease (radiation hepatitis) has a threshold of 30–35 Gy for the whole liver. Radiation-induced liver injury demonstrates an acute phase occurring within 3 months of exposure characterised by hyperaemia, venous congestion and fatty infiltration and a chronic phase with development of fibrosis and architectural distortion. On CT it appears as a sharply demarcated area of hypodensity corresponding to the radiation ports; however, in case of background fatty infiltration of liver, the irradiated area appears hyperdense compared to adjacent liver parenchyma. Radiation-induced pancreatic injury is characterised by parenchymal atrophy and fibrosis. The spleen is also radiosensitive and splenic atrophy occurs at doses of 35–40 Gy [41]. The upper poles of the kidneys (more commonly the left kidney because of its higher anatomical position than the right) can be affected by radiation. Radiation-induced cortical thinning and atrophy of the upper poles of the kidneys are well demarcated from the rest of the renal parenchyma and frequently seen on follow-up studies in patients with HL (Fig. 6) [42]. Uncommonly, lymphomatous involvement in the abdomen and pelvis can fistulise with the gastroitestinal tract resulting in tumour-bowel fistula (TBF). TBF can occur either spontaneously or following treatment with radiotherapy or chemotherapy. Gastrosplenic fistula can occur in association with gastric or splenic lymphoma and clinically manifests with epigastric pain and upper gastrointestinal bleeding [43, 44]. CT is the investigation of choice for the diagnosis of TBF with demonstration of a tract following oral contrast administration (Fig. 7).

Radiation therapy causes destruction of haematopoietic marrow elements and their replacement by fatty marrow elements usually occurring 3 to 6 weeks following radiotherapy; this is characterised by fatty replacement of marrow and manifests on MRI as T1 hyperintensity with a sharp demarcation corresponding to the radiation port [45]. Bone marrow recovery after radiation therapy can be reversible or irreversible depending on the amount of radiation given. Partial or complete recovery usually occurs at low doses (less than 30 Gy). Permanent fatty marrow replacement is usually seen above the dose of 36 Gy with very little chance of complete recovery [46].

## Complications of treatment

The main cause of increased mortality and morbidity among long-term survivors of HL are treatment-related development of secondary cancers, drug-related toxicities, radiation-induced pulmonary, cardiovascular and abdominal complications, thyroid disorders and myelosuppresion.

### Cardiovascular toxicity

**Fig. 8:**
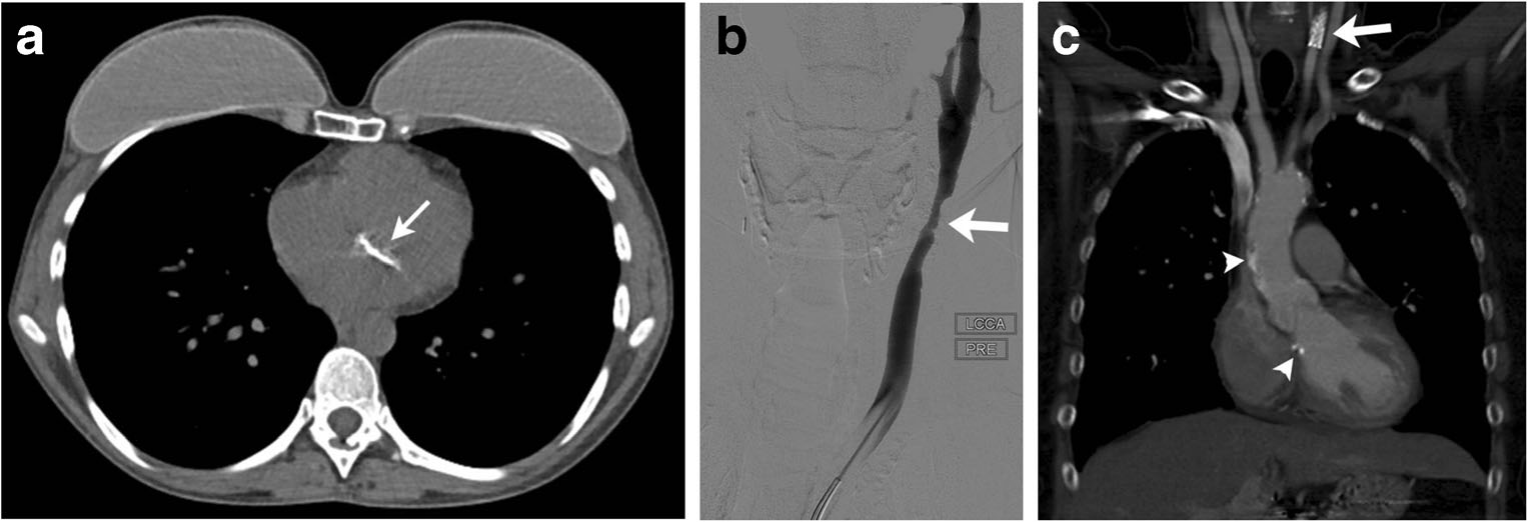
Cardiovascular complications. **a** A 38-year-old female with a history of nodular sclerosing HL at the age of 12 treated with chemotherapy and upper mantle radiation. Axial non-enhanced CT image shows mitral valve calcification (*arrow*). Note the bilateral mastectomies with a breast prosthesis from prior breast cancer. **b** A 40-year-old female with a history of nodular sclerosing HL at the age of 15 treated with chemotherapy and upper mantle radiation. Digital subtraction angiogram of the left carotid artery shows moderate to severe narrowing of the left distal common carotid artery (*arrow*). **c** Coronal reformatted image of contrast-enhanced CT during follow-up shows the presence of a stent in the left common carotid artery (*arrow*). Note the presence of aortic arch and right coronary artery calcifications (*arrowheads*). Also note the absence of spleen from the prior splenectomy

Cardiovascular toxicity in treated patients with HL can be due to doxorubicin and/or radiation. The cause-specific mortality for cardiovascular disease is approximately 10 % [47]. The cumulative incidence of adverse cardiac events in patients treated for HL is more than 20 % 25 years after radiotherapy and prior mediastinal radiation is a long-term risk factor for increased cardiovascular mortality and morbidity in HL patients, especially with doses above 30 Gy [48, 49]. Radiation produces dose-dependent cardiac damage through several different mechanisms. Radiation-induced cardiovascular complications can be acute, such as acute pericarditis, or chronic, such as coronary artery disease, valvular heart disease, decreased ventricular function, arrhythmia and chronic pericarditis. Initial evaluation of coronary artery disease is done with stress echocardiography or nuclear scintigraphy to look for inducible ischaemia. Patients with a positive stress test can be further evaluated with coronary CT angiography. Coronary calcifications are frequently visualised at follow-up chest CT in patients with treated HL (Fig. 8). Patients treated with radiation therapy are also at higher risk for development of noncoronary arteriosclerotic vascular disease including subclavian and carotid artery stenosis (Fig. 8). In a retrospective study of 414 patients treated with radiation therapy, Hull et al. found the incidence of noncoronary atherosclerotic disease was 2 % at 5, 3 % at 10 and 7 % at 20 years [50]. Radiation-induced pericardial disease can manifest in the form of pericarditis or pericardial effusion and is seen in 20 to 40 % of patients treated with mantle field radiation [51]. Echocardiography and MRI are useful modalities for the evaluation of pericardial disease. Radiation-induced valvular disease can manifest as both stenosis and insufficiency.

Anthracyclines cause dose-dependent cardiac toxicity that can produce irreversible cardiomyopathy. Left ventricular dysfunction has been reported to occur in 5 % of patients at cumulative doses as low as 240 mg/m^2^ [52]. Monitoring of left ventricular function with echocardiography, radionuclide angiography or less commonly cardiac MRI is recommended in patients who have received anthracycline-based chemotherapy. A multiple-gated acquisition (MUGA) scan is particularly useful for determination of the ejection fraction in HL patients before and after they undergo chemotherapy. A 10 % decline of the left ventricular ejection fraction (LVEF) below the lower limit of normal or an absolute decline of 45 % or decline of 20 % at any level is indicative of cardiac function deterioration. The risk of cardiac toxicity increases with combined treatment (doxorubicin and radiation) compared to either treatment given alone [48, 53, 54].

### Pulmonary toxicity

Like cardiovascular toxicity, pulmonary toxicity in HL patients can be due to radiotherapy or cytotoxic chemotherapy.

### Radiation-induced pulmonary toxicity

Newer radiotherapy techniques such as involved-site radiation therapy (ISRT) and intensity-modulated radiation therapy (IMRT) deliver less radiation to uninvolved normal organs compared to conventional involved-field or extended-field radiation therapy and reduce the long-term pulmonary and cardiovascular toxicity [55]. The degree of radiation damage depends on multiple factors such as the total radiation dose, fractionation, dose rate, volume of the lungs receiving a specified dose and beam arrangement [56].

Radiologic manifestations are usually confined to the lung tissue within the radiation port and are dependent on the interval after completion of treatment. Radiation-induced lung injury is divided into two clinical stages: the early stage characterised by radiation pneumonitis and late stage characterised by chronic radiation fibrosis. Early radiation pneumonitis usually occurs about 4–12 weeks after completion of radiation therapy and is characterised by ground-glass opacification, consolidation and atelectasis within the radiation portals. Radiation fibrosis usually develops within 6–12 months after completion of radiation therapy. Typical features of radiation fibrosis include a well-demarked area of volume loss, linear scarring or consolidation, parenchymal distortion and traction bronchiectasis, ipsilateral mediastinal shift and adjacent pleural thickening [34, 57].

### Bleomycin- and cyclophosphamide-induced pulmonary toxicity

**Fig. 9:**
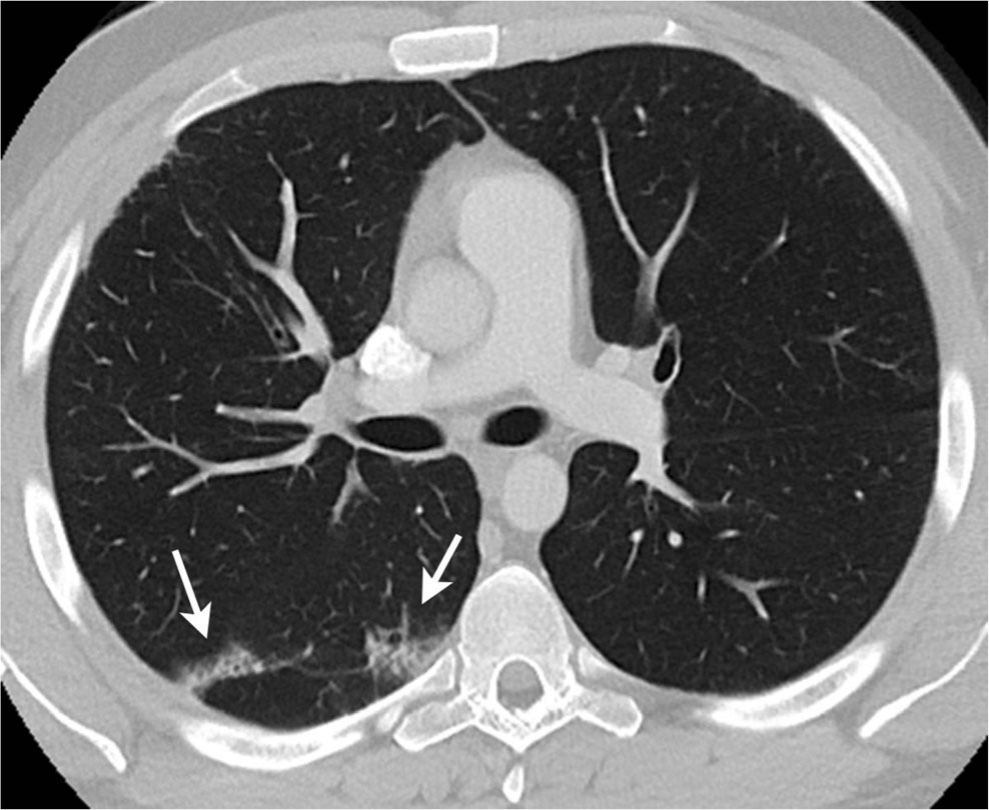
A 22-year-old male with nodular sclerosing HL on treatment with an ABVD regimen and complaining of new onset cough without fever. Axial contrast-enhanced lung window CT image shows patchy ground-glass opacities in the right lower lobe with peripheral subpleural distribution suggestive of drug toxicity

Bleomycin is commonly used in chemotherapy regimens of HL. Bleomycin-induced lung injury usually occurs in 3 %–20 % of treated patients [58]. HRCT is more sensitive than chest radiographs in identifying bleomycin-induced lung abnormalities. Common manifestations of bleomycin-induced lung disease are diffuse alveolar damage, nonspecific interstitial pneumonia (NSIP) and bronchiolitis obliterans organising pneumonia (BOOP) (Fig. 9). Cyclophosphamide can cause pulmonary haemorrhage and diffuse alveolar damage. CT manifestations of NSIP include patchy ground-glass opacities combined with reticular opacities in peripheral subpleural distribution. BOOP is characterised by bilateral asymmetrical ground-glass opacities or airspace consolidation in a peripheral or peribronchial distribution [59, 60].

### Second malignancies

**Fig. 10:**
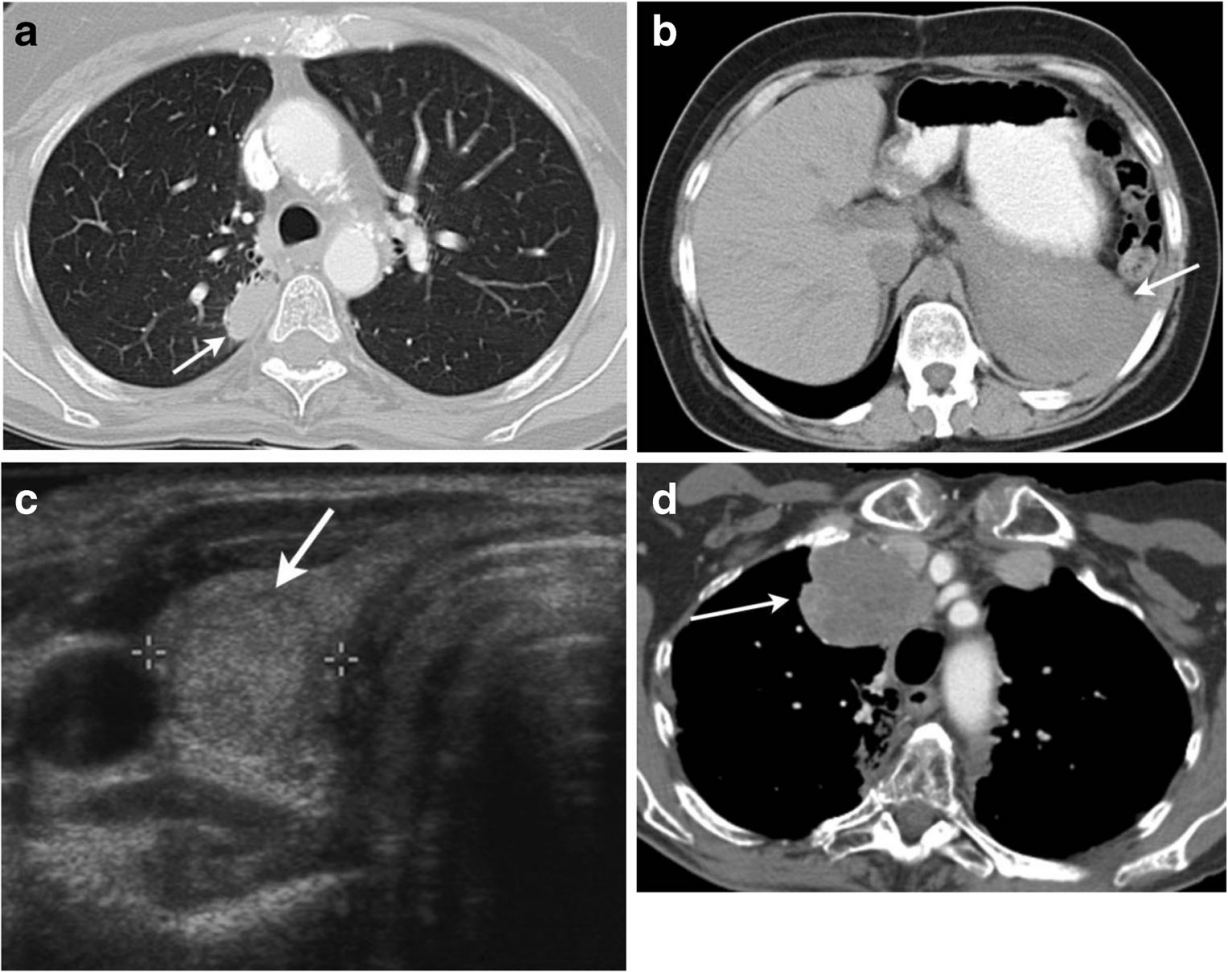
Second malignancies in patients with HL. **a** A 40-year-old female with a history of nodular sclerosing HL at the age of 15 treated with chemotherapy and upper mantle radiation. Axial contrast-enhanced lung window CT image shows a soft tissue mass within the right paramediastinal radiation field. Pathology was suggestive of adenocarcinoma of the lung. **b** A 24-year-old female presenting with upper abdominal pain and fullness with a prior history of HL at the age of 13 treated with splenectomy and radiation. Axial non-enhanced CT image of the upper abdomen shows a soft tissue mass lesion in the left upper abdomen (*arrow*). Pathology was suggestive of mesothelioma. **c** A 48-year-old female with a history of nodular sclerosing HL at the age of 15 treated with chemotherapy, mantle and pelvic radiation. Transverse greyscale ultrasound image shows a solid nodule in the right lobe of the thyroid (*arrow*). Fine-needle aspiration cytology from the nodule was suggestive of papillary thyroid carcinoma. **d** A 56-year-old female presenting with shortness of breath, intermittent cough and weight loss with a prior history of HL treated with radiation 40 years ago. Axial contrast-enhanced CT image shows a large heterogeneously enhancing anterior mediastinal mass (*arrow*). Pathology was suggestive of radiation-induced sarcoma

Second malignancies are the leading cause of death in long-term survivors of HL. Risk factors depend on the type of second malignancy and include the radiation dose and field, chemotherapy agents and doses administered, history of smoking and sex and age of the patient [61]. The maximum risk of a second cancer is at 5 to 9 years after chemotherapy alone, but after combined modalities the risk remains for 25 years or longer [62]. Solid tumours account for more than 50 % of second malignancies developing after 15 or more years of follow-up in patients treated for HL [63]. Lung and breast cancers are the most common secondary cancers in patients with HL due to their proximity to the radiation portal (Fig. 10a) [64]. Other solid tumours that have an increased incidence among HL survivors include gastrointestinal cancers, bone and soft tissue tumours, thyroid cancers, malignant melanoma, bladder cancer and malignant mesothelioma. The risk of developing secondary cancer is lower with combined modality treatment than with radiotherapy alone as the initial treatment [61, 63, 65, 66].

The risk of lung cancer is substantially increased in patients with HL treated with radiotherapy or chemotherapy, especially among smokers. The overall survival of non-small-cell lung cancer (NSCLC) in the setting of treated HL is inferior compared to patients with de novo lung cancer [67]. Breast cancers after HL typically occur after a long latency of 10–15 years and are associated with young age at irradiation. Annual breast screening (mammography or MRI) beginning no later than 8 to 10 years after treatment completion or at age 40 (whichever occurs earlier) is recommended for woman who have received chest or axillary radiation [68].

Patients with HL have a 20-fold increased risk of mesothelioma after radiotherapy. Mesothelioma arises from the mesothelial cells forming the serosal membranes of body cavities. Pleural and peritoneal cavities are frequent sites of involvement. Patients with lymphoma-associated malignant mesothelioma have younger age at diagnosis and longer overall survival compared with those with asbestos-associated mesothelioma [69]. CT is the primary imaging modality used for the evaluation of malignant pleural mesothelioma. Pleural mesothelioma presents as pleural thickening, pleural-based nodules, pleural effusion, thickening of interlobular fissures and pleural calcifications. The tumour frequently causes invasion of the chest wall, pericardium, heart and diaphragm. MRI is particularly helpful in detecting chest wall and mediastinal involvement because of the improved soft tissue contrast resolution [70, 71]. The CT features of malignant peritoneal mesothelioma are peritoneal- or omental-based masses as well as irregular or nodular peritoneal thickening with or without ascites (Fig. 10b) [72].

Radiotherapy is the main cause of thyroid cancers after HL, since the thyroid gland is a highly radiosensitive organ and is frequently exposed to radiation because of its location in neck (Fig. 10c). The risk of developing various thyroid abnormalities such as thyroid cancer, Grave’s disease, thyroiditis, hypothyroidism, nodular thyroid disease and thyroid atrophy is more than 50 % in patients with HL [40, 73, 74].

Patients with HL who received subdiaphragmatic radiotherapy have a dose-dependent increased risk of gastric cancer. The risk is further increased in patients who also received chemotherapy with MOPP (mechlorethamine, vincristine, procarbazine and prednisone) or BEACOPP regimes containing procarbazine. The stomach cancer risk is also increased in patients who are treated with dacarbazine, a component of the ABVD regimen, frequently used in patients with HL [75].

Radiation-induced sarcoma (RIS) is rare and associated with higher doses of radiation and with the use of concurrent chemotherapy (Fig. 10d). The mean latency period for post-radiation bone and soft tissue sarcomas ranges from 4 to 17 years [76]. Post-radiation sarcoma usually arises in or near the radiation field [77].

Imaging plays an important role in the timely detection of second malignancies in patients with HL. Knowledge of the history and site of prior radiotherapy can help the radiologists to increase the index of suspicion of second malignancies.

## Conclusion

In conclusion, although PET/CT remains the cornerstone for initial staging and response assessment at the completion of treatment in patients with HL, imaging modalities other than PET/CT often help in the initial workup of patients with HL. Treatment for HL usually depends upon the stage at diagnosis, histological subtype and prognostic factors, and chemotherapy or combination chemotherapy plus low-dose involved-field radiation therapy (LD-IFRT) is the standard treatment option in HL. Treatment response is assessed by restaging PET/CT using the Deauville criteria. Surveillance of HL patients after treatment is performed with plain radiographs and CT. Follow-up imaging can depict normal post-treatment changes or complications including second malignancies. Familiarity with expected post-treatment changes and complications on surveillance scans can help radiologists to guide patient management.

## References

[1] Jemal A, Siegel R, Ward E, Hao Y, Xu J, Thun MJ (2009). Cancer statistics, 2009. CA Cancer J. Clin.

[2] Campo E, Swerdlow SH, Harris NL, Pileri S, Stein H, Jaffe ES (2011). The 2008 WHO classification of lymphoid neoplasms and beyond: evolving concepts and practical applications. Blood.

[3] Vassilakopoulos TP, Angelopoulou MK, Siakantaris MP (2006). Pure infradiaphragmatic Hodgkin’s lymphoma. Clinical features, prognostic factor and comparison with supradiaphragmatic disease. Haematologica.

[4] Guermazi A, Brice P, de Kerviler EE (2001). Extranodal Hodgkin disease: spectrum of disease. Rad Rev Pub Radiol Soc North America, Inc.

[5] Siegel R, Naishadham D, Jemal A (2013). Cancer statistics, 2013. CA Cancer J. Clin.

[6] Hasenclever D, Diehl V (1998). A prognostic score for advanced Hodgkin’s disease. International prognostic factors project on advanced Hodgkin’s disease. N Engl J Med.

[7] Armitage JO (2010). Early-stage Hodgkin’s lymphoma. N Engl J Med.

[8] Aleman BM, Raemaekers JM, Tomisic R (2007). Involved-field radiotherapy for patients in partial remission after chemotherapy for advanced Hodgkin’s lymphoma. Int J Radiat Oncol Biol Phys.

[9] de Wit M, Bohuslavizki KH, Buchert R, Bumann D, Clausen M, Hossfeld DK (2001). 18FDG-PET following treatment as valid predictor for disease-free survival in Hodgkin’s lymphoma. Ann Oncol Off J Euro Soc Med Oncol/ESMO.

[10] Bednaruk-Mlynski E, Pienkowska J, Skorzak A (2015). Comparison of positron emission tomography/computed tomography with classical contrast-enhanced computed tomography in the initial staging of Hodgkin lymphoma. Leuk lymphoma.

[11] Juweid ME (2006). Utility of positron emission tomography (PET) scanning in managing patients with Hodgkin lymphoma. Hematol Edu Prog Am Soc Hematol Am Soc Hematol Educ Prog.

[12] Naumann R, Vaic A, Beuthien-Baumann B (2001). Prognostic value of positron emission tomography in the evaluation of post-treatment residual mass in patients with Hodgkin’s disease and non-Hodgkin’s lymphoma. Br J Haematol.

[13] Gallamini A, Rigacci L, Merli F (2006). The predictive value of positron emission tomography scanning performed after two courses of standard therapy on treatment outcome in advanced stage Hodgkin’s disease. Haematologica.

[14] Gallamini A, Hutchings M, Rigacci L (2007). Early interim 2-[18 F]fluoro-2-deoxy-D-glucose positron emission tomography is prognostically superior to international prognostic score in advanced-stage Hodgkin’s lymphoma: a report from a joint Italian-Danish study. J Clin Oncol Off J Am So Clin Oncol.

[15] Guay C, Lepine M, Verreault J, Benard F (2003). Prognostic value of PET using 18 F-FDG in Hodgkin’s disease for posttreatment evaluation. J Nuc Med Off Pub Soc Nuc Med.

[16] El-Galaly TC, Mylam KJ, Brown P (2012). Positron emission tomography/computed tomography surveillance in patients with Hodgkin lymphoma in first remission has a low positive predictive value and high costs. Haematologica.

[17] Girinsky T, Auperin A, Ribrag V (2014). Role of FDG-PET in the implementation of involved-node radiation therapy for Hodgkin lymphoma patients. Int J Radiat Oncol Biol Phys.

[18] Bradley AJ, Carrington BM, Lawrance JA, Ryder WD, Radford JA (1999). Assessment and significance of mediastinal bulk in Hodgkin’s disease: comparison between computed tomography and chest radiography. J Clin Oncol Off J Am Soc Clin Oncol.

[19] McInnes MD, Kielar AZ, Macdonald DB (2011). Percutaneous image-guided biopsy of the spleen: systematic review and meta-analysis of the complication rate and diagnostic accuracy. Radiology.

[20] Mayerhoefer ME, Karanikas G, Kletter K (2014). Evaluation of diffusion-weighted MRI for pretherapeutic assessment and staging of lymphoma: results of a prospective study in 140 patients. Clinical Cancer Res Off J Am Assoc Cancer Res.

[21] Stephane V, Samuel B, Vincent D (2013). Comparison of PET-CT and magnetic resonance diffusion weighted imaging with body suppression (DWIBS) for initial staging of malignant lymphomas. Eur J Radiol.

[22] Mayerhoefer ME, Karanikas G, Kletter K, et al. Evaluation of diffusion-weighted magnetic resonance imaging for follow-up and treatment response assessment of lymphoma: results of an 18 F-FDG-PET/CT-controlled prospective study in 64 patients. Clinical cancer research: an official journal of the American Association for Cancer Research 2015 10.1158/1078-0432.CCR-14-2454 25733598

[23] Carter BW, Wu CC, Khorashadi L (2014). Multimodality imaging of cardiothoracic lymphoma. Eur J Radiol.

[24] Wilson LD, Detterbeck FC, Yahalom J (2007). Clinical practice. Superior vena cava syndrome with malignant causes. N Engl J Med.

[25] Dahan H, Arrive L, Monnier-Cholley L, Le Hir P, Zins M, Tubiana JM (1998). Cavoportal collateral pathways in vena cava obstruction: imaging features. AJR Am J Roentgenol.

[26] Kapur S, Paik E, Rezaei A, Vu DN (2010). Where there is blood, there is a way: unusual collateral vessels in superior and inferior vena cava obstruction. Radiog Rev Pub Radiol Soc North Am Inc.

[27] Hirmiz K, Foyle A, Wilke D (2004). Intracranial presentation of systemic Hodgkin’s disease. Leuk lymphoma.

[28] Rabhi M, Ennibi K, Chaari J (2007). Toloune F [Hodgkin’s disease presenting with spinal cord compression]. Rev Neurol.

[29] Katabathina VS, Restrepo CS, Betancourt Cuellar SL, Riascos RF, Menias CO (2013). Imaging of oncologic emergencies: what every radiologist should know. Radiog Rev Pub Radio Soc North Am Inc.

[30] Engert A, Eichenauer DA, Dreyling M, Group EGW (2009). Hodgkin’s lymphoma: ESMO clinical recommendations for diagnosis, treatment and follow-up. Ann Onco Off J Euro Soc Med Oncol/ESMO.

[31] Smith MA, Seibel NL, Altekruse SF (2010). Outcomes for children and adolescents with cancer: challenges for the twenty-first century. J Clin Oncol Off J Am Soc Clin Oncol.

[32] Daw S, Wynn R, Wallace H (2011). Management of relapsed and refractory classical Hodgkin lymphoma in children and adolescents. Br J Haematol.

[33] Vargas HA, Hampson FA, Babar JL, Shaw AS (2009). Imaging the lungs in patients treated for lymphoma. Clin Radiol.

[34] Choi YW, Munden RF, Erasmus JJ (2004). Effects of radiation therapy on the lung: radiologic appearances and differential diagnosis. Radiog Rev Pub Radiol Soc North Am Inc.

[35] Apter S, Avigdor A, Gayer G, Portnoy O, Zissin R, Hertz M (2002). Calcification in lymphoma occurring before therapy: CT features and clinical correlation. AJR Am J Roentgenol.

[36] Strijk SP (1985). Lymph node calcification in malignant lymphoma. Presentation of nine cases and a review of the literature. Acta Radiol Diagn.

[37] Castellino RA (1992). Diagnostic imaging studies in patients with newly diagnosed Hodgkin’s disease. Ann Oncol Off J Euro Soc Med Oncol/ESMO.

[38] Nasseri F, Eftekhari F (2010). Clinical and radiologic review of the normal and abnormal thymus: pearls and pitfalls. Radiog Rev Pub Radiol Soc North Am Inc.

[39] Zhen Z, Sun X, Xia Y (2010). Clinical analysis of thymic regrowth following chemotherapy in children and adolescents with malignant lymphoma. Jpn J Clin Oncol.

[40] Brennan S, Hann LE, Yahalom J, Oeffinger KC, Rademaker J (2008). Imaging of late complications from mantle field radiation in lymphoma patients. Radiol Clin N Am.

[41] Kwek JW, Iyer RB, Dunnington J, Faria S, Silverman PM (2006). Spectrum of imaging findings in the abdomen after radiotherapy. AJR Am J Roentgenol.

[42] Izzedine H, Cluzel P, Deray G (2007). Renal radiation-induced arterial stenosis. Kidney Int.

[43] Tirumani SH, Baez JC, Jagannathan JP, Shinagare AB, Ramaiya NH (2013). Tumor-bowel fistula: what radiologists should know. Abdom Imaging.

[44] Dellaportas D, Vezakis A, Fragulidis G, Tasoulis M, Karamitopoulou E, Polydorou A. Gastrosplenic fistula secondary to lymphoma, manifesting as upper gastrointestinal bleeding. Endoscopy 2011; 43 Suppl 2 UCTN:E395 10.1055/s-0030-1256935 22275017

[45] Daldrup-Link HE, Henning T, Link TM (2007). MR imaging of therapy-induced changes of bone marrow. Eur Radiol.

[46] Casamassima F, Ruggiero C, Caramella D, Tinacci E, Villari N, Ruggiero M (1989). Hematopoietic bone marrow recovery after radiation therapy: MRI evaluation. Blood.

[47] Aleman BM, van den Belt-Dusebout AW, Klokman WJ, Van’t Veer MB, Bartelink H, van Leeuwen FE (2003). Long-term cause-specific mortality of patients treated for Hodgkin’s disease. J Clin Onco Off J Am Soc Clin Oncol.

[48] Galper SL, Yu JB, Mauch PM (2011). Clinically significant cardiac disease in patients with Hodgkin lymphoma treated with mediastinal irradiation. Blood.

[49] Hancock SL, Tucker MA, Hoppe RT (1993). Factors affecting late mortality from heart disease after treatment of Hodgkin’s disease. JAMA J Am Med Assoc.

[50] Hull MC, Morris CG, Pepine CJ, Mendenhall NP (2003). Valvular dysfunction and carotid, subclavian, and coronary artery disease in survivors of hodgkin lymphoma treated with radiation therapy. JAMA J Am Med Assoc.

[51] Adams MJ, Hardenbergh PH, Constine LS, Lipshultz SE (2003). Radiation-associated cardiovascular disease. Crit Rev Oncol/Hematol.

[52] Swain SM, Whaley FS, Ewer MS (2003). Congestive heart failure in patients treated with doxorubicin: a retrospective analysis of three trials. Cancer.

[53] Hodgson DC (2011). Late effects in the era of modern therapy for Hodgkin lymphoma. Hematol/Ed Prog Am Soc Hematol Am Soc Hematol Ed Prog.

[54] Busia A, Laffranchi A, Viviani S, Bonfante V, Villani F (2010). Cardiopulmonary toxicity of different chemoradiotherapy combined regimens for Hodgkin’s disease. Anticancer Res.

[55] Specht L, Yahalom J, Illidge T (2014). Modern radiation therapy for Hodgkin lymphoma: field and dose guidelines from the international lymphoma radiation oncology group (ILROG). Int J Radiat Oncol Biol Phys.

[56] Graves PR, Siddiqui F, Anscher MS, Movsas B (2010). Radiation pulmonary toxicity: from mechanisms to management. Semin Radiat Oncol.

[57] Park KJ, Chung JY, Chun MS, Suh JH (2000). Radiation-induced lung disease and the impact of radiation methods on imaging features. Radiograp Rev Pub Radiol Soc North Am Inc.

[58] Froudarakis M, Hatzimichael E, Kyriazopoulou L (2013). Revisiting bleomycin from pathophysiology to safe clinical use. Critical Rev Onco/Hematol.

[59] Rossi SE, Erasmus JJ, McAdams HP, Sporn TA, Goodman PC (2000). Pulmonary drug toxicity: radiologic and pathologic manifestations. Radiograp Rev Pub Radiol Soc North Am Inc.

[60] Cella L, Liuzzi R, D’Avino V (2014). Pulmonary damage in Hodgkin’s lymphoma patients treated with sequential chemo-radiotherapy: predictors of radiation-induced lung injury. Acta Oncol.

[61] Hodgson DC, Gilbert ES, Dores GM (2007). Long-term solid cancer risk among 5-year survivors of Hodgkin’s lymphoma. J Clin Oncol Off J Am Soc Clin Oncol.

[62] Swerdlow AJ, Higgins CD, Smith P (2011). Second cancer risk after chemotherapy for Hodgkin’s lymphoma: a collaborative British cohort study. J Clin Oncol Off J Am Soc Clin Oncol.

[63] Ng AK, Bernardo MV, Weller E (2002). Second malignancy after Hodgkin disease treated with radiation therapy with or without chemotherapy: long-term risks and risk factors. Blood.

[64] Nyandoto P, Muhonen T, Joensuu H (1998). Second cancer among long-term survivors from Hodgkin’s disease. Int J Radiat Oncol Biol Phys.

[65] Dores GM, Metayer C, Curtis RE (2002). Second malignant neoplasms among long-term survivors of Hodgkin’s disease: a population-based evaluation over 25 years. J Clin Oncol Off J Am Soc Clin Oncol.

[66] Franklin J, Pluetschow A, Paus M (2006). Second malignancy risk associated with treatment of Hodgkin’s lymphoma: meta-analysis of the randomised trials. Ann Oncol Off J Eur Soc Med Oncol/ESMO.

[67] Milano MT, Li H, Constine LS, Travis LB (2012). Variables affecting survival after second primary lung cancer: a population-based study of 187 Hodgkin’s lymphoma patients. J Thor Dis.

[68] Ng A, Constine LS, Advani R (2010). ACR appropriateness criteria: follow-up of Hodgkin’s lymphoma. Curr Probl Cancer.

[69] Chirieac LR, Barletta JA, Yeap BY (2013). Clinicopathologic characteristics of malignant mesotheliomas arising in patients with a history of radiation for Hodgkin and non-Hodgkin lymphoma. J Clin Oncol Off J Am Soc Clin Oncol.

[70] Wang ZJ, Reddy GP, Gotway MB (2004). Malignant pleural mesothelioma: evaluation with CT, MR imaging, and PET. Radiograp Rev Pub Radiol Soc North Am Inc.

[71] Yamamuro M, Gerbaudo VH, Gill RR, Jacobson FL, Sugarbaker DJ, Hatabu H (2007). Morphologic and functional imaging of malignant pleural mesothelioma. Eur J Radiol.

[72] Park JY, Kim KW, Kwon HJ (2008). Peritoneal mesotheliomas: clinicopathologic features, CT findings, and differential diagnosis. AJR Am J Roentgenol.

[73] Hancock SL, Cox RS, McDougall IR (1991). Thyroid diseases after treatment of Hodgkin’s disease. N Engl J Med.

[74] Shafford EA, Kingston JE, Healy JC, Webb JA, Plowman PN, Reznek RH (1999). Thyroid nodular disease after radiotherapy to the neck for childhood Hodgkin’s disease. Br J Cancer.

[75] Morton LM, Dores GM, Curtis RE (2013). Stomach cancer risk after treatment for Hodgkin lymphoma. J Clin Oncol Off J Am Soc Clin Oncol.

[76] Sheppard DG, Libshitz HI (2001). Post-radiation sarcomas: a review of the clinical and imaging features in 63 cases. Clin Radiol.

[77] O’Regan K, Hall M, Jagannathan J (2011). Imaging of radiation-associated sarcoma. AJR Am J Roentgenol.

